# CALENDAR

**DOI:** 10.1038/sj.bjc.6690109

**Published:** 1999-02


					15-18 February 1999
6th International Congress on Oral Cancer
New Delhi, India
Further information from:
Congress Secretariat, 6th International Congress on Oral Cancer,
509-B Sarita Vihar, New Delhi 110 044, India.
Tel: +91 11 694 4551, Fax: 91 11 694 4472
11-13 April 1999
MICRO 2000 International Microscopy Exhibition and
Conference
London, UK
Further information from:
Alison Winton, Exhibition Organizer, Royal Microscopical
Society, 37/38 St Clements, Oxford OX4 1AJ, UK.
E-mail: exhibitions@rms.org.uk
16 April 1999
Joint Meeting of the Section of Urology, Royal Society
of Medicine and the British Prostate Group - Prostate
Cancer: The Impact of Basic Science on Screening,
Treatment and Quality of Life
London, UK
Further information from:
Meeting Secretariat, c/o British Association of Urological
Surgeons, 35/43 LincolnOs Inn Fields, London WC2A 3PN, UK.
Tel: +44 (0) 171 405 1390, Fax: +44 (0) 171 404 5048
17-19 May 1999
Radiology 1999-Imaging, Science & Oncology
International Convention Centre, Birmingham, UK
Proferred papers are invited for poster presentation. Deadline for
Work in Progress: 22 February 1999
Further information from:
Radiology 1999 Secretariat, 36 Portland Place, London W1N 4AT,
UK. Tel: +44 (0) 171 307 1410, Fax: + 44 (0) 171 307 1414
Erratum
Chemotherapy for ovarian cancer - a concensus state-
ment on standard practice
M Adams, AH Calvert, J Carmichael, PI Clark, RE Coleman,
HM Earl, CJ Gallagher, TS Ganesan, ME Gore, JD Graham,
PG Harper, GC Jayson, SB Kaye, JA Lederman, RJ Osborne,
TJ Perren, CJ Poole, JA Radford, GJS Rustin, ML Slevin,
JF Smyth, H Thomas and PM Wilkinson
Br J Cancer 78(11): 14041406
The correct list of authors of this paper is reproduced below:
M Adams, RP A'Hern, AH Calvert, J Carmichael, PI Clark,
RE Coleman, HM Earl, CJ Gallagher, TS Ganesan, ME Gore,
JD Graham, PG Harper, GC Jayson, SB Kaye, JA Lederman,
RJ Osborne, TJ Perren, CJ Poole, JA Radford, GJS Rustin,
ML Slevin, JF Smyth, H Thomas and PM Wilkinson
CALENDAR
690
British Journal of Cancer (1999) 79(3/4), 690
(c) 1999 Cancer Research Campaign
Article no. bjoc.1998.0109

				

